# Predictive ability of a self-rated fall risk assessment tool in community-dwelling older women

**DOI:** 10.1007/s40520-023-02423-w

**Published:** 2023-05-05

**Authors:** Tommi Vilpunaho, Saija Karinkanta, Harri Sievänen, Juho Kopra, Heikki Kröger, Toni Rikkonen

**Affiliations:** 1 Kuopio Musculoskeletal Research Unit (KMRU), Surgery, Institute of Clinical Medicine, University of Eastern Finland (UEF), Yliopistonranta 1B, P.O. Box 1627, 70211 Kuopio, Finland; 2 The UKK Institute for Health Promotion Research, Tampere, Finland; 3 The Social Insurance Institution of Finland, Research Unit, Tampere, Finland; 4 Department of Orthopaedics, Traumatology and Hand Surgery, Kuopio University Hospital, Kuopio, Finland

**Keywords:** Accidental falls, Preventive medicine, Aged, Surveys and questionnaires, Self-assessment

## Abstract

**Background:**

Falls are a substantial health problem among older adults. An accessible and reliable tool for assessing individual fall risk is needed.

**Aims:**

The predictive ability of a one-page self-rated fall risk assessment form (KaatumisSeula® [KS]) was evaluated among older women in its current form.

**Methods:**

A subsample (n = 384) of community-living older women (aged 72–84 years) participating in the Kuopio Fall Prevention Study (KFPS) completed the KS form. Participants’ falls were prospectively registered for 12 months with SMS messages. Their group status and form-based fall risk category were compared to the verified fall events during the KFPS intervention. Negative binomial regression and multinomial regression analyses were used. Physical performance measurements (single leg stance, leg extension strength and grip strength) were used as covariates.

**Results:**

During the follow-up, 43.8% of women fell at least once. Among the fallers, 76.8% had at least one self-determined injurious fall, and 26.2% had falls requiring medical attention. According to KS, 7.6% of the women had low fall risk, 75.0% moderate, 15.4% substantial, and only 2.1% high fall risk. Women in the “moderate fall risk” group had 1.47-fold (95% CI 0.74–2.91; nonsignificant), in “substantial fall risk” 4.00-fold (1.93–8.3; p < 0.001) and in “high fall risk” 3.00-fold (0.97–9.22; nonsignificant) higher risk of falls compared to the “low fall risk” group. Performance in physical tests did not account for future falls.

**Conclusions:**

The KS form proved to be a feasible tool for self-administered fall risk assessment with moderate predictive ability.

**Trial registration:**

ClinicalTrials.gov identifier: NCT02665169, date of first registration 27/01/2016.

**Supplementary Information:**

The online version contains supplementary material available at 10.1007/s40520-023-02423-w.

## Background

Falls are a significant health issue causing fear of falls, fractures, and hospitalization. They are the second leading cause of unintentional injury deaths worldwide, especially among older adults [1]. In Finland, falls are the third most common cause of disability-adjusted life years [2]. In 2019, fall-related injuries caused over half of all accidental deaths in Finland. Around 90% of these persons were over 65 years old [3]. The number of fall-induced deaths has more than doubled in Finland during the past 40 years, mostly due to ageing of the population [4]. The proportion of older people is increasing rapidly, and it is predicted that in the near future the number of fall-induced deaths may increase 1.6-fold and 1.5-fold higher in Finnish men and women, respectively [5]. A recent global recommendation suggests that even among “healthier” older adults the fall risk should be reassessed annually [6]. Thus, a reliable tool for practical assessment of older persons’ fall risk is needed.

Numerous fall risk assessment tools, such as questionnaires and computer-based algorithms, are available. Some of these tools are designed to be used for hospitalized persons [7–14], whereas others [15–18] are intended for community-dwellers. However, most of these tools are not routinely used among community-living older people. Some tools are composed of more than 20 items [16, 19, 20], and although comprehensive, they are not quick to use. Moreover, many tools require data from clinical measurements of weight [21], grip strength [21, 22], walking [21, 22] and/or balance [21–25]. Furthermore, scoring from symptom scales such as MMSE or CES-D is often required [22, 26]. Some rapid tools for fall risk assessment and screening, such as FROP-Com [18], are available, but they rely on evaluation performed by health care professionals. Only a few tools consist of a simple self-rating for older adults [17, 27, 28].

In the present study, we assessed the predictive ability of a one-page self-rated fall risk assessment tool (KaatumisSeula^®^ [KS]) among older women included in the Kuopio Fall Prevention Study (KFPS) during a 12-month follow-up. Results of physical performance measurements (single leg stance, leg extension strength, and grip strength) were used as covariates. The assessment was made according to existing KS risk categories with no intention to readjust the cut-off points or methodological aspects of the KS itself.

## Methods

### KaatumisSeula^®^

The UKK Institute for Health Promotion Research (Tampere, Finland) recently released the KaatumisSeula^®^ (KS, meaning “falls screen” in English) self-evaluation tool for assessing the fall risk of older people and implementing preventive measures in the community [29, 30]. The one-page assessment form [31] is designed to be filled in either by the older persons themselves or together with (health care) professionals and used as a simple first line risk assessment for falls among general population even when older people together discuss the fall risk and how it could be managed. Moreover, the KS can also be used as an early-stage assessment in other situations where one’s fall risk is a concern. The KS also includes written material of falls prevention for the older adults.

The items of the KS tool are based on the relevant scientific literature on established factors affecting the fall risk in older adults and expert opinions working in the field of fall prevention. The form consists of six multiple-choice questions concerning age, fall history, balance and movement confidence, independence of daily living, chronic health conditions, and physical activity. Each question adds 0 to 2 or 4 points, and the total score ranges from 0 to 14 points. Based on the score, respondents are classified into four categories: (1) “Your fall risk is not elevated” (0 points), (2) “Your fall risk is elevated” (1–5 points), (3) “Your fall risk is clearly elevated. *A professional assessment is recommended”* (6–8 points), and (4) “Your fall risk is great. *A professional assessment is required”* (9–14 points) (Online Appendix 1). In this article, the categories were renamed as follows: (1) low fall risk (0 points); (2) moderate fall risk (1–5 points); (3) substantial fall risk (6–8 points); and (4) high fall risk (9–14 points). The form has also been released as an electronic version in Finnish [32]. Test–retest reliability of the KS has been tested among older people (n = 13) who participated in 1 week course of the Finnish Pensioners’ Federation. Test–retest reliability was very good for total score (kappa = 0.906) and good for fall risk classification (kappa = 0.755) [33].

### KFPS study and participants

The Kuopio Fall Prevention Study (KFPS) was a 2-year exercise randomized controlled trial (RCT) conducted in 2016–2019. It evaluated the effects of physical exercise, including gym training and Tai Chi, on the prevention of falls and promotion of well-being in older women [34]. The study cohort consisted of 914 women aged 72–84 years and living in the city of Kuopio in Finland. Participants were randomized to intervention (n = 457) and control groups (n = 457). The present KS assessment study consisted of subsample of 384 KFPS women.

During the study, the participants received biweekly Short Message Service (SMS) questions concerning falls (during the past 2 weeks) with a simple “yes/no” option to answer. Positive replies (yes) were verified for details by means of a phone interview. In addition, participants filled a self-administered diary with falls and leisure-time physical activity at 3-month intervals. National health care registers were cross-checked for fractures. Both study groups had physical performance measurements three times: at baseline and at the 12-month and 24-month follow-ups. The protocol of the KFPS study is available for further details [34].

### Assessment of the KS fall risk assessment tool

The KS form was introduced to a subsample of 403 women during the 12-month study visit, which was set as the baseline of this study. Falls were followed up for the second year of the KFPS. Altogether, a subsample of 384 women from the KFPS (220 from the intervention group and 164 from the control group) both with successfully filled the KS forms and sufficient follow-up time after the 12-month study visit (mean follow-up 369 days, range 308–390 days) were included in the analysis. The participants did not get the results (i.e., estimated fall risk category) of their KS forms during the study. The clinical characteristics of the study participants are shown in Online Appendix 2.

Participants’ group status and fall risk category based on the KS were compared to all falls and injurious falls. Moreover, physical performance measurements (i.e., single-leg stance, maximal leg extension strength and maximal grip strength) [34] were made during the 12-month visit of the KFPS. The proportions of fallers were calculated for each KS score as follows: the number of women who had fallen during the follow-up was divided by the total number of women (both fallers and non-fallers) within each KS score. The original KS risk categories were used without readjusting the cut-off points. However, raw data features of falls per KS score is available for those interested (Online Appendix 3).

### Statistical analysis

Negative binomial regression was used to examine how the KS fall risk category predicted falls during the 12-month follow-up period. Group status, follow-up time, single-leg stance time, leg extension strength and grip strength were used as covariates. The three physical performance measures served as independent predictors of the statistical model. The 95% confidence intervals of the regression coefficients are presented. Poisson log-linear regression was used to examine how well the KS risk points predicted the probability of future falls. Multinomial logistic regression was used in the sub-analysis to examine whether the intervention group status or fall risk category affected the incidence of falls leading to an injury. P values less than 0.05 were considered statistically significant. Statistical analyses were performed with SPSS version 27.

## Results

### The fall risk categories of the KFPS participants and falls during the follow-up

**Fig. 1:**
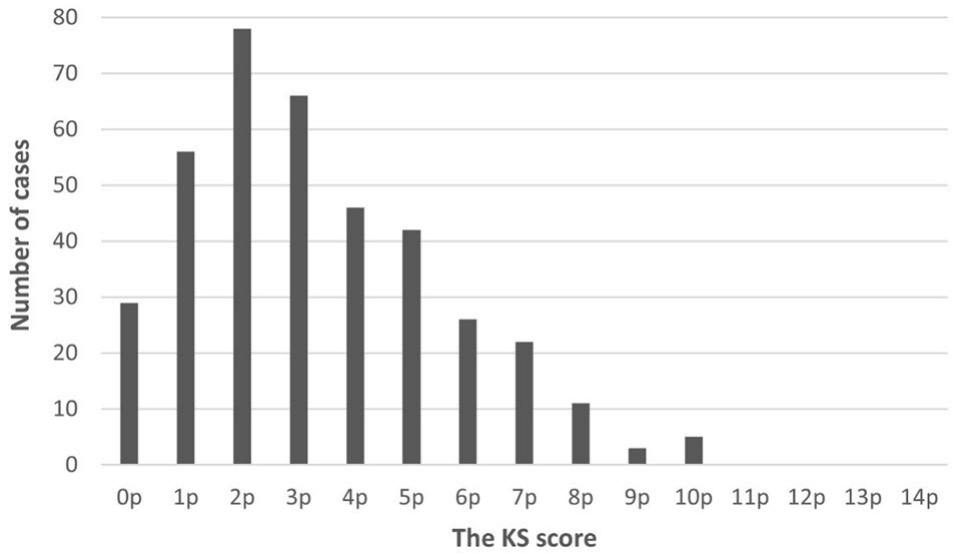
Distribution of the KS scores among the participants (N = 384). None of the participants had the highest scores from 11 to 14 points. 0 points = low fall risk, 1–5 points = moderate fall risk, 6–8 points substantial fall risk, 9–14 points = high fall risk

Among the 384 women, the KS indicated “low” fall risk for 29 (7.6%) women, “moderate” for 288 (75.0%), “substantial” for 59 (15.4%), and “high” only for 8 (2.1%). The highest KS score was 10 points in five women: none had the highest scores from 11 to 14 points (Fig. 1).

During the 12-month follow-up, 168 women out of 384 (43.8%) fell at least once, and the total number of falls was 309. Most fallers had only a few registered falls (on average 0.8 fall for each participant), but two women (0.5%) had up to 13 falls (Table 1). Of the women who had fallen, 126 (75%) had at least one self-determined fall injury and 44 cases (26.2% of fallers) required medical attention (Table 1).

**Table 1:** The number of falls during the 12-month follow-up

Total number of falls	Participants (n = 384)
0	216 (56.3%)
1	109 (28.4%)
2	27 (7.0%)
3	12 (3.1%)
4	10 (2.6%)
5	6 (1.6%)
6	1 (0.3%)
8	1 (0.3%)
13	2 (0.5%)
Number of fall events leading to injury	
0	258 (67.2%)
1	96 (25.0%)
2	16 (4.2%)
3	9 (2.3%)
4	4 (1.0%)
6	1 (0.3%)
Number of fall events requiring medical attention	
0	340 (88.5%)
1	39 (10.2%)
2	5 (1.3%)

### Predictive value of the KS

Negative binomial regression showed that the group status (intervention/control) of the KFPS was not significantly associated with the KS predictions when analyzing all falls (Table 2). Altogether, a higher KS score predicted a higher incidence of falls. Initially, we studied how the continuous KS score would explain number of falls in log-linear Poisson regression adjusted with intervention indicator and found out that each one-point increment in KS score would yield exp(B) to be 1.25 (95% CI 1.17–1.34, p < 0.001). As a categorical predictor, women in the “moderate fall risk” group had a 1.47-fold (95% CI 0.74–2.91, p = 0.27) higher risk for falls during the next 12 months compared to women with “low fall risk”. Correspondingly, women in the “substantial fall risk” category had a 4.00-fold (95% CI 1.93–8.30, p < 0.001), and in the “high fall risk” group a 3.00-fold (95% CI 0.97–9.22, p = 0.056) higher risk for falls during the next 12 months compared to the “low fall risk” group. Overall, using analysis of deviance test (Chi-squared = 27.2773, df = 1, p < 0.001) the KS classification variable was found to be a crucial predictor in the model which also had the intervention variable as a predictor. The inclusion of single-leg stance time, leg extension and grip strength as covariates did not change the results of the negative binomial regression. None of the three physical performance variables were significantly associated with overall fall incidence and thus they were omitted from the final model (Table 2). Three women had a substantially higher number of falls (eight or more) in comparison to the others. Analyses were also made without these outliers, which did not affect the results and therefore they were included in the final model.

**Table 2:** Negative binomial regression analysis of 384 women with 309 falls for the 12-month follow-up

			Exp (B)^a^	95% CI^b^	p value^c^
Crude values^d^					
Study group					
Intervention			0.84	0.61–1.14	0.26
Control			1		
Fall risk category	Falls per person	Injury per fall			
High fall risk (9–14p)	1.38	0.82	3.00	0.97–9.22	0.056
Substantial fall risk (6–8p)	1.73	0.53	4.00	1.93–8.30	< 0.001
Moderate fall risk (1–5p)	0.64	0.58	1.47	0.74–2.91	0.27
Low fall risk (0p)	0.45	0.54	1		
Adjusted values^d^					
Study group					
Intervention			0.84	0.61–1.15	0.27
Control			1		
Fall risk category					
High fall risk (9–14p)			3.36	0.92–12.2	0.067
Substantial fall risk (6–8p)			3.77	1.77–8.04	0.001
Moderate fall risk (1–5p)			1.52	0.76–3.05	0.237
Low fall risk (0p)			1		
Covariates					
Single leg stance (seconds)^e^			0.995	0.980–1.010	0.49
Leg extension strength (Newtons)^f^			1.002	1.000–1.004	0.095
Grip strength (Newtons)^g^			1.003	0.999–1.008	0.13

^a^Exp(B) = Exponentiated values of the coefficients

^b^CI = 95% confidence interval

^c^p value < 0.05 was considered statistically significant

^d^Results are shown for the crude values and for adjusted model with physical performance results as covariates

^e^Eyes open with the better foot, time measured continuing for a maximum of 30 s

^f^Mean of the best result for both legs from three attempts

^g^Handheld dynamometer (Jamar, Sammons-Preston, Illinois, USA) the dominant hand with three attempts, the best result was used in the analysis

In the sub-analysis concerning the injuries, belonging to the higher fall risk group increased the risk of a fall-related injury. Women with “substantial fall risk” and “high fall risk” had 5.93-fold (95% CI 2.03–17.3, p = 0.001) and 8.71-fold (95% CI 1.34–56.8, p = 0.024) higher risks for injurious falls than women in the “low fall risk”, respectively. Neither the intervention group status nor physical performance was significant in this sub-analysis (Table 3).

**Table 3:** Multinomial logistic regression analysis for falls with injury

	Exp(B)^a^	CI^b^	p value^c^
Fall(s) with injury^d^			
Study group			
Intervention	1.07	0.68–1.70	0.77
Control	1		
Fall risk category			
High fall risk (9–14p)	8.71	1.34–56.8	0.024
Substantial fall risk (6–8p)	5.93	2.03–17.3	0.001
Moderate fall risk (1–5p)	1.69	0.66–4.35	0.28
Low fall risk (0p)	1		
Fall(s) with no injury^d^			
Study group			
Intervention	1.04	0.53–2.04	0.91
Control	1		
Fall risk category			
High fall risk (9–14p)	5.24	0.32–86.6	0.25
Substantial fall risk (6–8p)	4.44	0.84–23.6	0.080
Moderate fall risk (1–5p)	1.88	0.42–8.46	0.41
Low fall risk (0p)	1		

^a^Exp(B) = Exponentiated values of the coefficients

^b^CI = 95% confidence interval

^c^p value < 0.05 was considered statistically significant

^d^The reference category is “No fall events with injury”

**Fig. 2:**
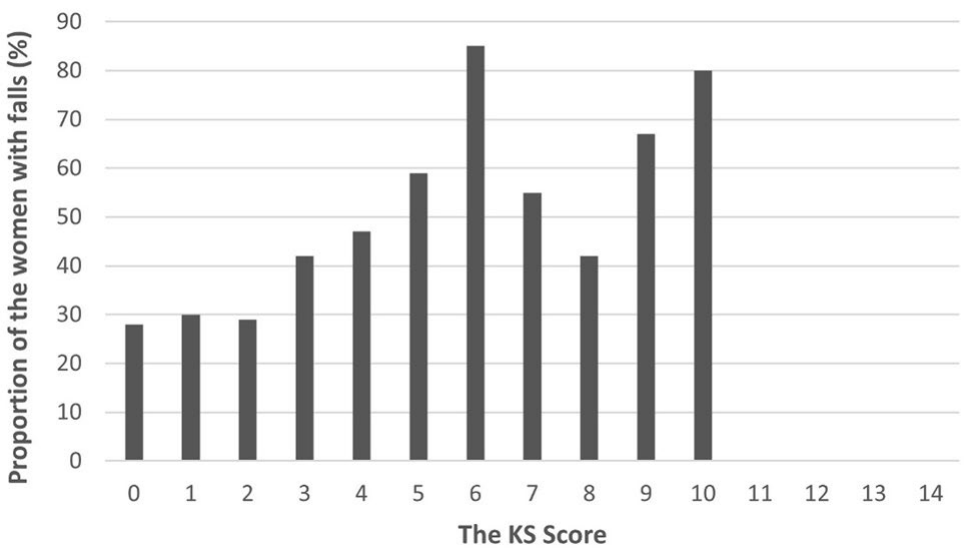
Proportions of fallers within KS scores during the follow-up year. The proportion was calculated by dividing the number of women with fall(s) having a certain score by the total number of all women (fallers and non-fallers) with the same KS score. None of the women had the highest scores of 11–14

The proportion of fallers increased with a higher KS score (Fig. 2). However, this association was non-linear, as the highest proportion was seen with a score of six, which is a middle-range score in the form. According to the fall history, the women with a score of six (n = 26) had also reported more falls prior to the KS fall assessment compared to all other scores (Chi-square test, p < 0.001) which appears to be in line with the highest incidence of falls during the follow-up. The definition of the cut-off scores for the KS fall risk groups among the KFPS population would not be apparent, as the increase in the proportion of both fallers and falls was non-linear in the highest scores above six (Online Appendix 3).

## Discussion

This prospective 12-month study among community-living older women examined not only the ability of a Finnish self-rated fall risk assessment tool KaatumisSeula^®^ (KS) to predict falls but also the utility of additional physical performance measurements in parallel with the KS score. The KS was shown to be a feasible tool for fall risk prediction, whereas the physical performance measurements provided no added value.

Although the risk was elevated with more than 90% of the participants, around half of them did not fall during the follow-up. As age is a major risk factor for falls, all 75-year-old persons are assigned at least one point in the KS. Therefore, most of the participants were categorized in the “moderate fall risk” group (1–5 points). As previous falls are a risk factor for future falls, a person with one fall in the past 12 months receives two points, and a person with ≥ 2 falls four points in the KS. Of those women who fell, three out of four hurt themselves to some degree, and approximately every fourth faller required medical attention. In the sub-analysis, the KS was able to predict injurious falls in the “substantial” (almost six times higher risk for injurious fall) and “high” fall risk (almost ninefold higher risk) groups compared to the “low fall risk” group.

In terms of overall fall risk, the KS was able to predict falls in the “substantial fall risk” (fourfold higher risk for falls) and in the “high fall risk” (threefold higher risk, non-significant) groups compared to the “low fall risk” group. However, based on the proportions of fallers, the women with lower scores appeared to have a relatively higher risk for future falls than those with the highest scores. One explanation could be that older women with more risk factors for falls may self-adjust their daily actions in risk-reducing manners. The small number of women (n = 8) in the “high fall risk” group also makes the analyses in this category uncertain. Nevertheless, six points in the KS is the cut-off recommending “professional assessment”, which appears to be in line with the results. The KS could not significantly predict fall risk in the “moderate fall risk” group. However, it is worth noting that even with the lowest KS scores of 0–2, almost 30% of the women fell during the follow-up, with a KS score of 3 over 40% of the women fell, and with 5 points more than half of the women fell. According to Poisson regression, fall risk increased almost 23% per each increased KS point.

Adjustment with physical performance did not affect the results. This was expected, because questions on balance, safety in movement and physical activity are already included in the KS, and good performance in these areas correlates with sufficient physical capability (e.g., good lower limb strength). On the other hand, physically inactive older people may be less likely to fall because of spending less time on their feet. Previous results of the KFPS showed that more active older women may have an increased risk for falls and fractures [35]. Other studies have also suggested that physically more active older people may have more fall events [36]. Physical activity may even increase the risk for upper limb fractures [37, 38]. However, multi-component exercise RCTs have indicated long-term reductions in injurious falls, falls requiring medical attention, and fractures among older Finnish women [39–41].

The other simple self-rated fall risk evaluation tool for older adults have had similar results as the KS. For example, a short postal screening tool to prevent falls injury (PreFIT) had only modest predictive ability of any falls (AUC 0.66), recurrent falls (AUC 0.70) as well as fractures (AUC 0.60) in a large UK study among community dwelling older adults [28]. Moreover, a 13-item self-fillable Fall Risk Questionnaire (FRQ) performed well (kappa = 0.875, p < 0.001) when comparing to clinical evaluation, although some items of the tool had only moderate correlation with the clinical exam in a small study [27]. Also, an Activities-specific balance confidence scale (ABC) could predict future falls well (p = 0.003 in linear regression) during a 6-month follow-up among a small cohort of community dwelling older adults. However, it could correctly classify the fall status only in around 78% of the participants [17]. Similar findings were also stated in a recent meta-analysis observing that a single tool is not able to detect older people’s falls with sufficient accuracy, whereas the use of two or more assessment tools in combination may enhance the predictability of falls [14]. Thus, in future studies, the outcomes of the self-administered KS could be verified further with clinically measured tests for falls screening, such as Timed Up & Go test and Berg Balance scale [14]. However, as discussed above, physical performance assessments alongside the KS would not necessarily improve fall prediction. Also, a more throughout evaluation of the KS would require having more participants with the highest fall risk.

The strength of this study is its relatively large sample size and the intensive follow-up protocol utilizing biweekly SMS questions and the verification of falls with phone interviews. Since several participants with notable fall history sustained multiple falls also during the follow-up, a simple “yes/no” interpretation was an easy and adequate way to report fall events.

The participants of the KFPS study are known to be physically and mentally healthier than the non-participating and non-invited women within the region [35]. This participation bias, along with the lack of men, limits the generalizability of the results at the population level. Furthermore, as the participants were community-living older women, the results cannot be extrapolated to people living in residential care or nursing homes. Also, during the KFPS intervention the participants were not in their habitual routine which may influence the results. Only 17.5% of the women had substantial or high fall risk according to the KS in the present study, and none of the participants had the highest scores from 11 to 14 points. This underrepresentation of the “most frail” women with the highest scores was expected due to demographic bias among this study based on voluntary participation. Thus, whether the incidence of falls is truly greatest in the high fall risk group remains to be studied with other cohorts.

The original KS risk categories were used in this study without any intention to modify the methodological aspects of the KS. A left-skewed distribution of the KS scores was evident and even if the risk factors of falls are well-established, apparently the cutoff points are subject to change. However, the redefinition of the KS categories would not be appropriate by utilizing the KFPS population alone, as the cut-off values of the KS would presumably be different in other populations.

The present study provided relevant information on issues that may need revision in the further versions of the KS fall risk assessment tool, and further research on this topic is still needed. However, it seems that the KS has future development potential for a simple, self-administered screening tool evaluating the risk of falls among the general population of older people. The World Falls Guidelines Task Force recently recommended that “opportunistic case finding for falls risk is recommended for community-dwelling older adults” [6] and KS may become an effective tool to answer this need.

## Conclusions

The KS tool showed moderate reliability in predicting falls among this cohort of older women. However, the optimal number and location of cutoff points needs further research and validation.

## Supplementary Information

Below is the link to the electronic supplementary material.

Supplementary file1 (DOCX 21 kb)

## Data Availability

The raw data of the current study includes personal information and therefore it is not available as such. However, the data can be de-identified and made available on reasonable request from the corresponding author. The code used may also be made available upon request.
