# Supplementary material for: Predictive ability of a self-rated fall risk assessment tool in community-dwelling older women
Source: Aging Clin Exp Res. 2023 May 5;35(6):1205–12. doi: 10.1007/s40520-023-02423-w (PMC10200780; doi:10.1007/s40520-023-02423-w)
Supplement: Supplementary file 1 — Supplementary file1 (DOCX 21 kb) [file 40520_2023_2423_MOESM1_ESM.docx]

**Article title:** Predictive ability of a self-rated fall risk assessment tool in community-dwelling older women

**Journal name:** Aging Clinical and Experimental Research

**Author names:** Tommi Vilpunaho, Saija Karinkanta, Harri Sievänen, Juho Kopra, Heikki Kröger, Toni Rikkonen

**Affiliation and e-mail address of the corresponding author:** Tommi Vilpunaho, Kuopio Musculoskeletal Research Unit (KMRU), Yliopistonranta 1b, P.O. Box 1627, 70211 Kuopio, Finland. E-mail address: tommi.vilpunaho@uef.fi

# APPENDIX 1.

Questions of the KaatumisSeula self-evaluation form and interpretation of the total score in four fall risk categories.

| **Questions** | **Choices** | **Points** |
| --- | --- | --- |
| Question 1:  What age group do you belong to? | Under 75 years  75-84 years  85 years or older | 0 p  1 p  2 p |
| Question 2:  Have you fallen in the past 12 months? | No  Yes, once  Yes, 2 or more times | 0 p  2 p  4 p |
| Question 3:  Do you think your balance is good and do you feel safe when moving? | Yes, I feel safe without any aids  Yes, with aids  No, I feel unsafe about my balance and/or movement | 0 p  1 p  2 p |
| Question 4:  Do you need help in coping with daily chores and activities? | No, I can handle everything myself  Yes, I need some help with some chores  Yes, I need a lot of help | 0 p  1 p  2 p |
| Question 5:  Do you have any of these conditions?^1^ | No  Yes, one  Yes, two or more | 0 p  1 p  2 p |
| Question 6:  How often do you engage in physical activity? | 3 times a week or more for at least 30 min at a time  1-2 times a week for at least 30 min at a time  Occasionally, or not at all | 0 p  1 p  2 p |
|  |  |  |
| **Interpretation of the total score** |  |  |
| 0 points | Low fall risk | |
| 1-5 points | Moderate fall risk | |
| 6-8 points | Substantial fall risk. A professional assessment is recommended | |
| 9-14 points | High fall risk. A professional assessment is required | |

^1^ Heart disease, diabetes, Parkinson’s disease, vertigo, osteoporosis, impaired vision, memory disorder, stroke, respiratory disease, sensory loss in the lower limbs, musculoskeletal disorder (e.g. osteoarthritis, joint replacement in lower limbs).

# APPENDIX 2.

KFPS baseline characteristics of the women who completed the KS form.

| **Characteristic** | **Intervention group (n=220)** | **Control group (n=164)** | **p-values** |
| --- | --- | --- | --- |
| Age ± SD^1^; years | 75.8 ± 3.0 | 75.7±2.8 | F=0.60  p=0.75 |
| Body mass index ± SD; kg/m^2^ | 27.8 ± 4.6 | 27.4±4.4 | F=0.19  p=0.38 |
| Subjective health; % |  |  |  |
| Very good | 5.0 | 6.1 |  |
| Good | 45.5 | 46.0 |  |
| Moderate | 47.3 | 46.0 | χ^2^=0.34 |
| Poor or very poor | 2.2 | 1.9 | p=0.95 |
| Subjective physical condition; % |  |  |  |
| Very good | 4.1 | 3.7 |  |
| Good | 39.1 | 45.1 |  |
| Moderate | 44.5 | 42.1 | χ^2^=1.84 |
| Poor or very poor | 12.3 | 9.1 | p=0.61 |
| Present ambulatory status; % |  |  |  |
| Fully mobile | 59.4 | 64.4 |  |
| Mobile, not able to run | 36.9 | 33.1 |  |
| Able to walk max 1000 meters | 3.2 | 2.5 | χ^2^=1.69 |
| Able to walk max 100 meters | 0.5 | 0 | p=0.64 |
| Fall(s) in last 12 months; % |  |  |  |
| Yes | 40.4 | 43.5 | χ^2^=0.37 |
| No | 59.6 | 56.5 | p=0.54 |
| Fear of falling; % |  |  |  |
| Often or always | 7.3 | 6.7 |  |
| Sometimes | 23.2 | 20.1 |  |
| Seldom | 29.1 | 33.5 | χ^2^=1.06 |
| No fear | 40.5 | 39.6 | p=0.79 |
| Marital status; % |  |  |  |
| Unmarried | 7.3 | 6.1 |  |
| Cohabitation or married | 47.9 | 48.8 |  |
| Divorced | 18.7 | 20.7 | χ^2^=0.51 |
| Widow | 26.0 | 25.3 | p=0.92 |
| Living arrangements; % |  |  |  |
| Alone | 50.0 | 52.4 |  |
| With partner | 47.7 | 46.3 | χ^2^=0.72 |
| With children or other person(s) | 2.3 | 1.3 | p=0.70 |

^1^Standard deviation

# APPENDIX 3.

Features of the study population per KaatumisSeula score.

| **Score of the KaatumisSeula** | **Fall injuries per person** | **Proportion of injuries** | **Number of women** | **Proportion of fallers ^1^** | **Falls per person ^2^** |
| --- | --- | --- | --- | --- | --- |
| **0** | 0.24 | 0.21 | 29 | 0.28 | 0.45 |
| **1** | 0.2 | 0.2 | 56 | 0.30 | 0.35 |
| **2** | 0.31 | 0.24 | 78 | 0.29 | 0.38 |
| **3** | 0.41 | 0.27 | 66 | 0.42 | 0.60 |
| **4** | 0.39 | 0.33 | 46 | 0.47 | 1.17 |
| **5** | 0.64 | 0.48 | 42 | 0.59 | 1.00 |
| **6** | 1.0 | 0.73 | 26 | 0.85 | 2.04 |
| **7** | 0.86 | 0.41 | 22 | 0.55 | 1.18 |
| **8** | 0.82 | 0.36 | 11 | 0.42 | 1.75 |
| **9** | 1.0 | 0.33 | 3 | 0.67 | 1.33 |
| **10** | 1.2 | 0.8 | 5 | 0.80 | 1.20 |

1Number of fallers having a certain score divided by the number of all the women having that score

^2^Total number of fall events among women having a certain score divided by the number of all the women having that score
